# Direct effects of nitrogen addition on seed germination of eight semi‐arid grassland species

**DOI:** 10.1002/ece3.6576

**Published:** 2020-07-20

**Authors:** Tong Zhang, Mengzhou Liu, Xudong Huang, Wei Hu, Ning Qiao, Hongquan Song, Bing Zhang, Rui Zhang, Zhongling Yang, Yinzhan Liu, Yuan Miao, Shijie Han, Dong Wang

**Affiliations:** ^1^ International Joint Research Laboratory of Global Change Ecology School of Life Sciences Henan University Kaifeng China; ^2^ College of Environment and Planning Henan University Kaifeng China; ^3^ College of Water Resources North China University of Water Resources and Electric Zhengzhou China; ^4^ Key Laboratory of Mollisols Agroecology Northeast Institute of Geography and Agroecology Chinese Academy of Sciences Harbin China; ^5^ Key Laboratory National Forestry Administration on Ecological Hydrology and Disaster Prevention in Arid Regions Northwest Surveying, Planning and Designing Institute of National Forestry and Grassland Administration Xi'an China

**Keywords:** atmospheric nitrogen deposition, germination rate, plant diversity, plant functional group

## Abstract

Seed germination plays an important role in mediating plant species composition of grassland communities under nitrogen (N) enrichment. Shifts of plant community structure with N‐enhanced deposition in terrestrial ecosystems have occurred globally. Despite numerous studies about the effects of enhanced N deposition on mature plant communities, few studies have focused on seed germination. Using a laboratory experiment, we report the effects of five N concentrations, including 0, 5, 10, 20, and 40 mM N (NH_4_NO_3_) on seed germination of eight semi‐arid grassland species. Results showed that low N concentrations (5‐ and 20‐mM N) promoted mean final germination proportion of all eight species by 4.4% and 6.4%, but high concentrations (40 mM N) had no effect. The mean germination rate was decreased 2.1% and 5.1% by higher N concentration (20‐ and 40‐mM N) levels, but germination start time showed the opposite trend, delayed by 0.7, 0.9, and 1.8 d for the 10, 20, and 40 mM N treatments. Final germination proportion, mean germination rate, and germination start time were significantly different among species in response to N concentration treatments. The final germination proportion of *Allium tenuissimum* and *Chenopodium glaucum* were suppressed by increased N concentration, whereas it increased for *Potentilla bifurca, Plantago asiatica,* and *Setaria viridis*. Our findings provide novel insights into N deposition‐induced species loss based on seed germination factors in semi‐arid grassland communities.

## Graphical abstract

Low N concentrations promoted mean final germination proportion of all species. The responses of eight species to N addition were differences. Mean germination rate was lower at higher N concentration levels.

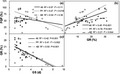

## 1 INTRODUCTION

Nitrogen deposition resulting from human activities has become a global environmental issue (Chen et al., 2019; Deng et al., 2017; Liang et al., 2020; Liu et al., 2013). The availability of nitrogen is a critical control on plant diversity and growth in many terrestrial ecosystems, including grasslands in Northern China (Bobbink et al., 2010; Wang et al., 2020). Plant diversity is mediated at different stages of plant growth, including seed germination, seedling growth, age of senescence, and offspring performance. Most studies focus on the responses of mature or offspring plant species diversity to N deposition (Bobbink et al., 2010; Hu & Wan, 2019; Liu et al., 2019; Xia & Wan, 2013; Zhou et al., 2019). It is also necessary to study how natural seed germination directly responds to N deposition.

Seed germination is the first crucial stage of plant maturation, affecting the growth and development of individual plants, thereby affecting community dynamics (Dürr, Dickie, Yang, & Pritchard, 2015; Jia, Chen, Fan, Li, & Zhang, 2020). The proportion of seeds germinating and the germination timing affect species establishment and colonization success and play a critical role in promoting species coexistence within communities. Since seed germination determines when and where seedling growth begins, the success of seedling establishment depends on seed germination (Tobe, Zhang, & Omasa, 2005). Many plants have dormancy mechanisms that suppress seed germination until environmental conditions are favorable (Adondakis & Venable, 2004). Thus, proper timing and final proportion of seeds germinating are important for the survival and propagation of plants (Mérai et al., 2018) and may also reflect environmental conditions under which a given species is most likely to succeed (Brändle, Stadler, Klotz, & Brandl, 2003).

Seed germination is controlled by several internal (seed size, nutrient content, and phylogeny) and external (temperature, moisture, oxygen, available nutrients, pH, and salinity) factors (Bu et al., 2007; Kolodziejek, Patykowski, & Wala, 2017; Norden et al., 2009; Osuna, Prieto, & Aguilar, 2015; Yang et al., 2019). Under favorable conditions, seeds absorb water and exhibit radicle protrusion, but under others, they may become dormant or die (Miransari & Smith, 2014). Nitrogen deposition is likely more crucial than other abiotic variables in determining suitable conditions for a plant (Bobbink et al., 2010; Clark et al., 2007). Nitrogen is an essential nutrient and many plants in terrestrial ecosystems are adapted to conditions of low N availability; in addition to serving as a basic nutrient, N also promotes seed germination through function as a signaling molecule (Osuna et al., 2015). Yet, high N availability may suppress seed germination and plant development by changing the levels of metal ions, abscisic acid, phytochromes, or seed water absorption (Chen et al., 2020; Grubišić & Konjević, 1990; Sun, Wang, He, & Hao, 2018; Tian et al., 2016; Yan et al., 2016). Furthermore, the negative effects of N deposition on plant diversity may be depended on plant functional group, and result in loss of perennial forbs, or annuals and biennials (Gao, Wang, Fu, & Zhao, 2017; Zhong, Miao, Han, & Wang, 2019). However, few studies have investigated how N deposition affects seed germination of different functional groups, especially under variable N deposition levels in semi‐arid grassland ecosystems.

The grasslands in Northern China are a critical component of the Eurasian steppe, providing important ecological and economic services for sustainable development (Kang, Han, Zhang, & Sun, 2007; Miao, Liu, et al., 2020). The amount of N deposition is increasing due to increased human activity, posing a threat to plant diversity in the semi‐arid region (Liu et al., 2013; Tian et al., 2016; Wang et al., 2020). This study aimed to investigate the effects of N deposition on seed germination of two functional groups (perennial forbs, four species; annuals and biennials, four species). The following questions were posed: (a) Does seed germination respond to N deposition? and (b) How does N deposition affect seed germination of two functional groups?

## 2 MATERIALS AND METHODS

### 2.1 Study site and seed selection

Our experimental seeds were collected from a temperate steppe of Inner Mongolia, Northern China (42°02′N, 116°17′E, 1, 324 m a. s. l.). The long‐term mean annual precipitation is 385.5 mm, with ~ 90% distributed May–October. The mean annual temperature is 2.1°C, with a range from −17.5°C in January to 18.9°C in July. According to FAO classification, the soil type is Haplic Calcisols, with an average of 16.95% clay, 20.30% silt, and 62.75% sand. Four perennial forbs (*Artemisia frigida, Allium tenuissimum, Potentilla tanacetifolia, Potentilla bifurca*) and four annuals and biennials (*Chenopodium aristatum, Plantago asiatica, Chenopodium glaucum, Setaria viridis*) those co‐occur in the grassland were selected (Miao, Xuan, et al., 2020; Sagar, Li, Singh, & Wan, 2019; Song, Niu, & Wan, 2016). Plant seeds were collected in the field in September 2015 and germination occurred in November 2015.

### 2.2 Germination experiments

The experiments were conducted in an automatic greenhouse at Henan University. Germination experiments were conducted in 9 cm diameter plastic Petri dishes on 2 layers of filter paper. Distilled water was added until seeds floated, but they were not inundated. Seed germination of all species was evaluated under five N concentrations: 0, 5, 10, 20, and 40 mM N (NH_4_NO_3_). Each N treatment level had 3 replicates of 50 seeds. NH_4_NO_3_ is a common source of N in grassland experiments. The emergence of the radicle was the criterion for germination (Lai et al., 2019). Germination of seeds was monitored daily over 30 days, and germinated seeds removed promptly to allow for more likely germination of other seeds (Lai et al., 2019).

### 2.3 Statistical analyses

Germination was measured using three indices: final germination proportion (FGP), germination rate (GR), and germination start (GS).

The FGP is the proportion of sown seeds that germinated (Lai et al., 2019).

GR is calculated as follows:

$$\text{GR}=\sum 100G_{i}/nt_{i}$$

where *G_i_* is the number of seed germinated on day *t_i_* (*t_i_* = 0, 1, 2, 3, …) and *n* is the number of seeds used in an experiment (Daws et al., 2002). Higher GR values represent more rapid seed germination.

The GS is time (day) between seed sowing and the start of germination (Nasr & Shariati, 2005).

Two‐way analysis of variance (ANOVA) was used to determine the statistical significance of N treatment, species, and their interactions on FGP, GR, and GS. The Duncan's post hoc tests were used for multiple comparisons when a significant effect was detected. One‐way ANOVA was used for multi‐comparisons of the effect of N concentration on FGP, GR, and GS of each species, followed by Duncan's post hoc tests. A linear model (*y* = *a* + *bx*) was used to determine the relationship between pairs of FGP, GR, and GS. All statistical analyses were performed using the R statistical software environment (R Core Team 2018, version 3.5.2).

## 3 RESULTS

Two‐way ANOVA showed that both N addition and species had a significant effect on final germination (FGP), germination rate (GR), and starting time (GT). Moreover, there was an interaction effect of N and species on FGP, GR, and GT (Table 1). *A. frigida* had the highest GP (95.3) and GR (28.1). *C. glaucum* had the earliest GT. *P. bifurca* had the lowest FGP (16.1) and GR (1.9), and the latest GT (5.3).

**TABLE 1:** Results (*F*‐values) of two‐way ANOVAs on the effects of nitrogen (N), species (SP), and their interactions on final germination (FGP), germination rate (GR), and germination start (GS)

Source of variation	FPG	GR	GS
SP	172.7***	161.7***	62.3***
N	4.4**	14.4***	23.3***
SP*N	3.8***	5.8***	8.8***

** *p* < .01.

*** *p* < .001.

### 3.1 Effects of nitrogen concentration on final germination proportion

**FIGURE 1:**
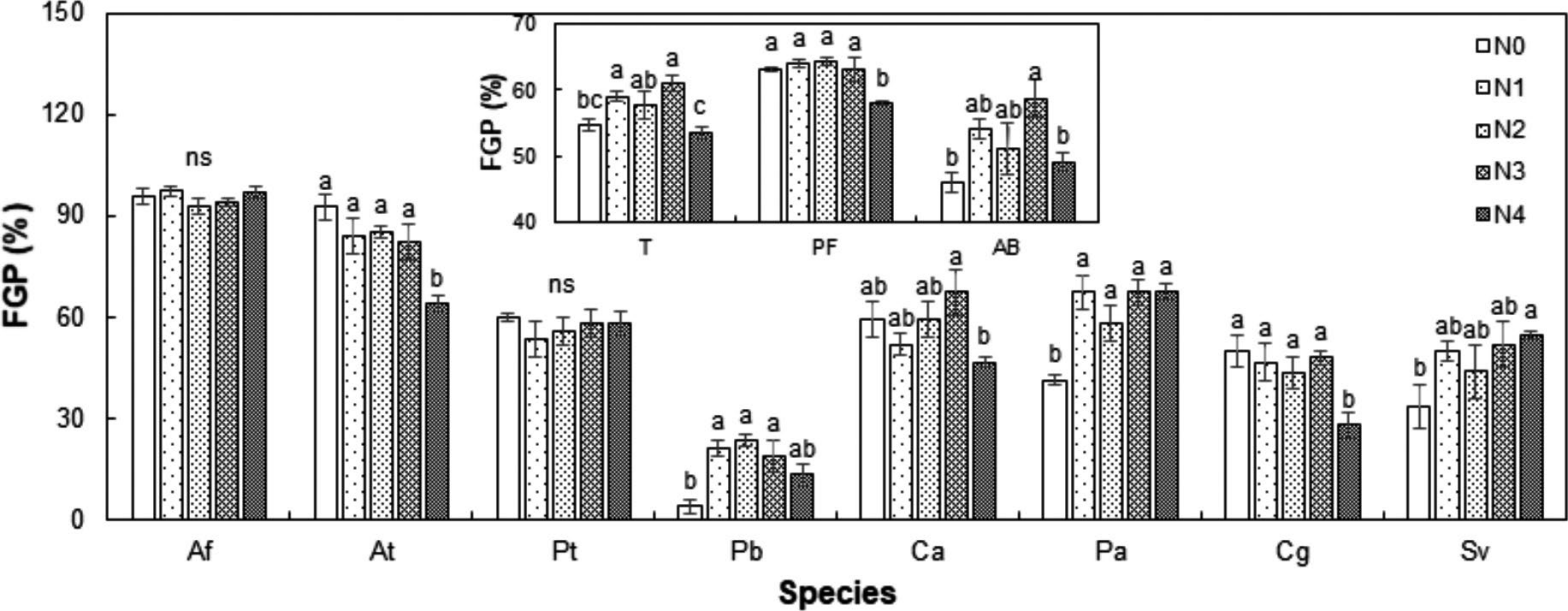
Effects of nitrogen (N) concentration on final germination proportion (FGP) all species, perennial forb (PF), annuals and biennials (AB), and each species individually. Different letters over the bars suggest significant differences among treatments based on Duncan's multiple range tests (*p* < .05). Af: *A. frigida*; At: *A. tenuissimum*; Pt: *P. tanacetifolia*; Pb: *P. bifurca*; Ca: *C. aristatum*; Pa: *P. asiatica*; Cg: *C. glaucum*; Sv: *S. viridis*

Nitrogen treatment had a significant effect on mean FGP of all species, as well as perennial forb (PF), and annuals and biennials (AB) functional groups (all *p* < .05, Figure 1 inset). Compared to the control, mean FGP was enhanced 4.4% and 6.4% (absolute change) under N1 and N3 treatments, whereas mean FGP of the PF functional group was suppressed by 5.1% under the N4 treatment and mean FGP of the AB functional group was enhanced by 12.7% under the N3 treatment (all *p* < .05; Figure 1). When analyzed by species, N treatment had a significant effect on FGP of *A. tenuissimum, P. bifurca, P. asiatica,* and *C. glaucum*. Compared to the control, FGP of *A. tenuissimum* was suppressed by 28.7% under the N4 treatment, and FGP of *P. bifurca* was enhanced by 17.3%, 19.3%, and 14.6% under the N1, N2, and N3 treatments, respectively. FGP of *P. asiatica* was enhanced by 26.0%, 16.7%, 26.0%, and 26.0% under the N1, N2, N3, and N4 treatments, respectively, and FGP of *C. glaucum* was suppressed by 22.0% under the N4 treatment (Figure 1).

### 3.2 Effects of nitrogen concentration on germination rate

**FIGURE 2:**
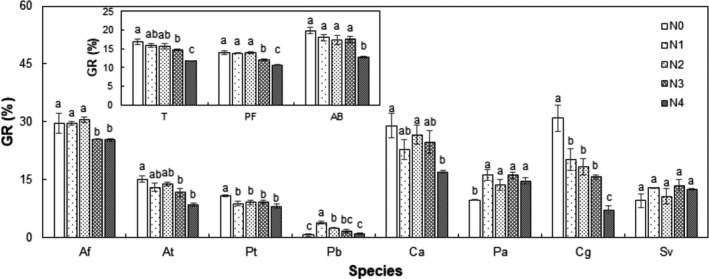
Effects of nitrogen (N) concentration on germination rate (GR) of all species, perennial forb (PF), annuals and biennials (AB), and each species individually. Different letters over the bars show significant differences among treatments based on Duncan's multiple range tests (*p* < .05). Species abbreviations are given in Figure 1

Nitrogen treatment had a significant effect on the mean GR of all species, as well as PF and AB functional groups (Table 1, Figure 2). Compared to the control, mean GR of all species were suppressed by 2.1% and 5.1% (absolute change) under the N3 and N4 treatments, mean GR of PF functional group was suppressed by 2.0% and 3.3% under the N3 and N4 treatments, and mean GR of AB functional group was suppressed by 7% under the N4 treatment (all *p* < .05; Figure 2). When analyzed by species, N treatment had a significant effect on GR of all species, except *S. viridis* (Figure 2). Compared to the control, we found the following: GR of *A. frigida* was suppressed by 4.2% and 4.3% under the N3 and N4 treatments; GR of *A. tenuissimum* was suppressed by 3.4% and 6.1% under the N3 and N4 treatments; GR of *P. tanacetifolia* was suppressed by 2.1%, 1.8%, 1.6%, and 2.8% under the N1, N2, N3, and N4 treatments; GR of *P. bifurca* was enhanced by 3.1% and 1.7% under the N1 and N2 treatments; GR of *C. aristatum* was suppressed by 12% under the N4 treatment; GR of *P. asiatica* was enhanced by 6.5%, 4.0%, 6.4%, and 4.9% under the N1, N2, N3, and N4 treatments; and GR of *C. glaucum* was suppressed by 10.7%, 12.6%, 15.3%, and 23.7% under the N1, N2, N3, and N4 treatments, respectively (Figure 2).

### 3.3 Effects of nitrogen concentration on the germination start time

**FIGURE 3:**
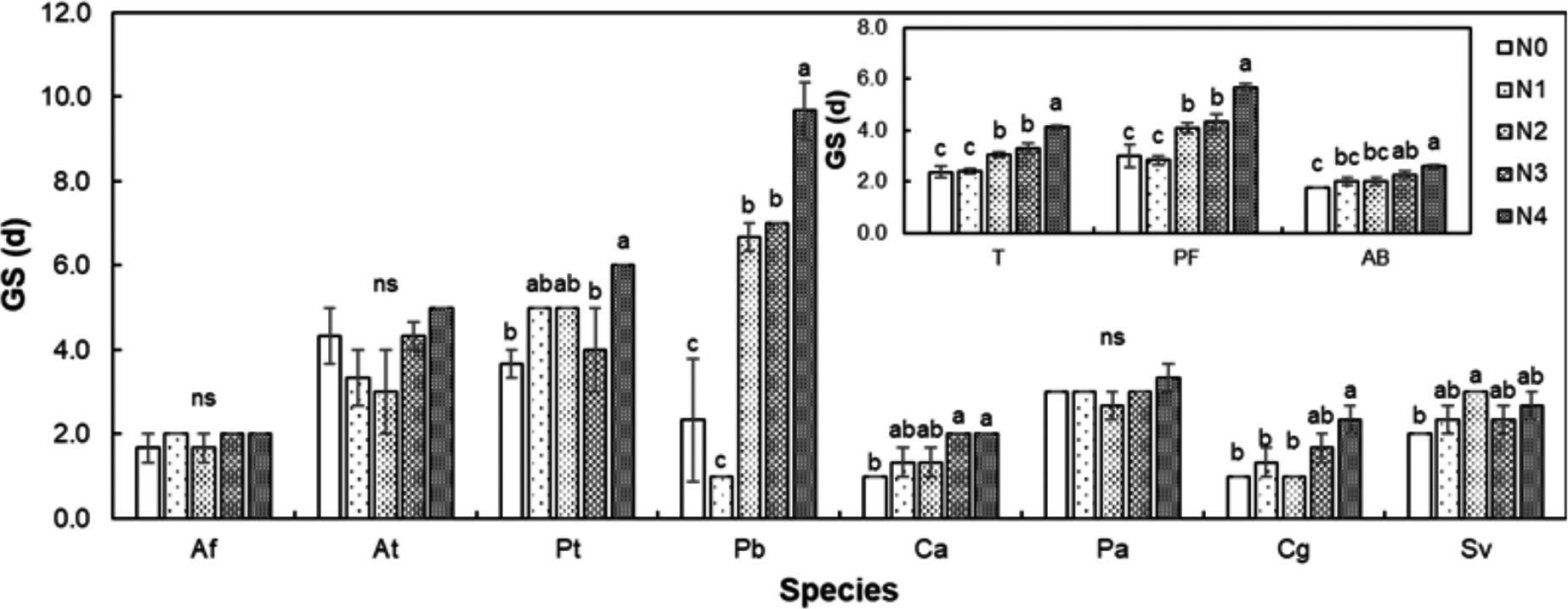
Effects of nitrogen concentration on germination start (GS) of all species, perennial forb (PF), annuals and biennials (AB), and each species individually. Different letters over the bars show significant differences among treatments based on Duncan's multiple range tests (*p* < .05). Species abbreviations are given in Figure 1

Nitrogen treatment had a significant effect on GS of all species, as well as for PF and AB functional groups (all *p* < .05, Figure 3). Compared to the control, results include the following: mean GS of all species was delayed by 0.7, 0.9, and 1.8 d under the N2, N3, and N4 treatments; mean GS of the PF functional group was delayed by 1.1, 1.3, and 2.7 d under the N2, N3, and N4 treatments; and mean GS of the AB functional group was delayed by 0.5 and 0.8 d under the N3 and N4 treatments, respectively (all *p* < .05; Figure 3). When analyzed individually, the N treatment had a significant effect on GS of *P. tanacetifolia*, *P. bifurca*, *C. aristatum*, and *C. glaucum* (Figure 3). Compared to the control, results included: GS of *P. tanacetifolia* was delayed 2.3 d under the N4 treatment; GS of *P. bifurca* was enhanced by 4.3, 4.7, and 7.3 d under the N2, N3, and N4 treatments; GS of *C. aristatum* was delayed by 1 d under both the N3 and N4 treatments; GS of *C. glaucum* was delayed 1.3 d under the N4 treatment; and GS of *S. viridis* was delayed 1 d under the N4 treatment (Figure 3).

### 3.4 Relationships of final germination proportion, germination rate, and germination start

**FIGURE 4:**
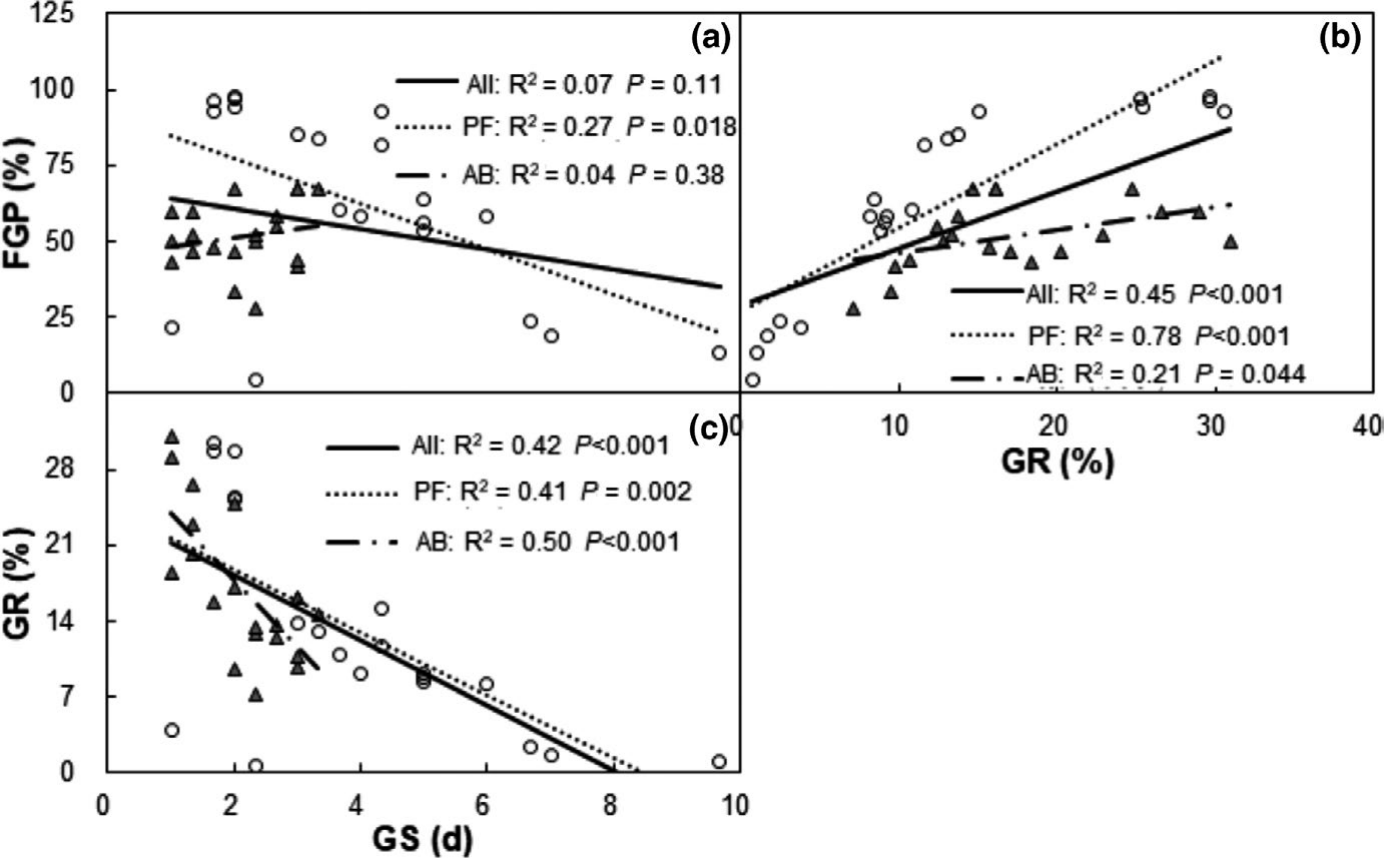
Relationships among mean final germination proportion (FGP), mean germination start (GS), and mean germination rate (GR). Each data point represents the mean value of each species under each treatment

Across treatments, mean FGP of all species and the PF functional group were negatively correlated with mean GS, but that was not the case for the AB functional group (Figure 4a). Mean FGP of all species and PF and AB functional groups showed a positive relationship with mean GR (Figure 4b). Mean GR of all species, the PF and AB functional groups had a negative relationship with mean GS (Figure 4c).

## 4 DISCUSSION

This study demonstrates that N deposition can significantly affect seed germination of semi‐arid plants. Along an increasing N concentration gradient, mean FGP of all species had a hump‐shaped pattern, mean GR decreased, and mean GS increased. The FGP, GR, and GS were significantly different between PF and AB, as well as among the eight species individually.

In our study, the mean FGP of all species showed a hump‐shaped pattern for the focal species. The optimal concentration of the nitrogenous solutions for germination was 10 mM, whereas higher concentrations had a lower effect (Kolodziejek et al., 2017). We found that low nitrogen concentration promoted seed germination, such as for *P. bifurca*, *P. asiatica*, and *S. viridis*, with no negative effect under high nitrogen concentrations. In contrast, the mean FGP of *A. tenuissimum* and *C. glaucum* decreased under the highest N concentrations. Nitrogen can be used by plants not only as a nutrient, but it also acts as a germination signal (Tiansawat & Dalling, 2013; Yan et al., 2016). A previous study found that reduction of abscisic acid levels is controlled in a nitrate‐dependent manner, specifically by proteins (which are transcription factors) binding to a promoter of a gene coding an abscisic acid catabolic enzyme (Wang et al., 2015; Yan et al., 2016). In another study, nitrogen addition enhanced germination in 9 of 53 species representative of the flora in Central‐Eastern Spain (Luna & Moreno, 2009); the optimal nitrogen concentration seems to promote germination by lowering the abscisic acid/gibberellins ratio (Song, Xiang, et al., 2016; Yan et al., 2016). Nitrates that naturally occur in the soil can override light requirements in some cases (Daws et al., 2002). The positive responses of nitrogen on seed germination are related to phytochromes (Grubišić & Konjević, 1990). Nitrate may enhance the number of Pfr‐receptors or may act as a Pfr cofactor (Grubišić & Konjević, 1990). However, higher nitrogen concentrations can also result in a toxic effect on seed germination for certain species in specific environmental contexts (Pérez‐Fernández, Calvo‐Magro, Montanero‐Fernández, & Oyola‐elasco, 2006). The sensitivity of different species germination to nitrogen addition is various (Davis, 2007). Previous studies found that the N application could delay the seed germination rate of *Bromus inermis* (Zhu, Wang, Yan, Mao, & Mao, 2018). Therefore, the seed germination responses to N treatments were species‐specific, mainly positive or unimodal (Ochoa‐Hueso & Manrique, 2010).

We also found that higher nitrogen reduced mean GR of all species, as well as for the PF and AB functional groups. The GR of five species were reduced at higher concentrations, but not for one PF (*P. bifurca*) and two AB (*P. asiatica* and *S. viridis*). Moreover, higher nitrogen significantly delayed the mean GS of all species, as well as for the PF and AB functional groups. Water uptake is a fundamental requirement for the initiation and completion of seed germination (Huang et al., 2020). Increased nitrogen concentration can slow or inhibit seed water absorption, inhibiting GR and GS (Wen et al., 2017). Differences in germination time and related variables can help optimize long‐term success by increasing the probability that seedlings will emerge and grow under more favorable environmental conditions (Rice & Dyer, 2001). Correlation analysis showed that mean FGP was negatively correlated with GS of PF, but not correlated with GS of AB. This finding suggests species that can germinate early may have a competitive advantage, especially for perennial plants. However, many annuals and biennials have a bet‐hedging strategy for germination and germinate at very high rates under suitable environmental conditions (Gremer & Venable, 2014).

Resources utilization varied greatly among the plant functional groups (Wang, Zhang, Zhu, Yang, & Li, 2018). Variation in strategies of seed germination among species may contribute to coexistence at a community level, because it allows for temporal partitioning of resources, as well as providing a buffer against local species extirpation (Chesson, 2000). In the process of seed germination, other environmental factors also affect FGP, such as temperature and water potential (Lai et al., 2019). Correlation analysis showed that the mean GR of seed germination was negatively correlated with GS in all species and the two functional groups (Figure 4). Seedlings that rapidly establish would have an advantage when there is a competition for resources.

Nitrogen deposition slows seed germination, seedling establishment, and plant diversity in many terrestrial ecosystems (Li, Wen, Hu, & Du, 2010; Varma, Iyengar, & Sankaran, 2016; Zhong et al., 2019). However, many laboratory experiments, including the present study, have shown that low levels of nitrogen addition can have positive effects on seed germination (Tiansawat & Dalling, 2013; Yan et al., 2016). Loss of species diversity caused by nitrogen deposition may not be caused by the direct effect of nitrogen concentration. Instead, previous field studies on species loss have shown that light asymmetry, the toxicity of metal ions, and competition (even though all are affected by nitrogen deposition) are more important than nitrogen concentration alone (DeMalach, Zaady, & Kadmon, 2017; Tian et al., 2016). For example, in one study N deposition increased mobilization of soil Mn, with a 10‐fold greater accumulation of Mn in forbs than in grasses, resulting in a reduction of forbs abundance (Tian et al., 2016). Nitrogen addition can also increase the concentration of ferric iron and aluminum in soils through ion exchange processes, that can be toxic to seeds and seedlings (Liu, Zhang, & Lal, 2016; Roem, Klees, & Berendse, 2002). Light asymmetry reduces the abundance of light‐adapted species under the condition of nutrient enrichment (DeMalach et al., 2017). Nitrogen addition can indirectly affect the composition of soil microbial and animal communities (Kim et al., 2015; Shao et al., 2017; Zhao et al., 2018), which may lead to degradation of seeds by microorganisms (Chee‐Sanford, Williams, Davis, & Sims, 2006). Our results show the role of nitrogen in seed germination, providing new insights into species loss under various N addition scenarios.

## 5 CONCLUSIONS

Our findings showed that N enrichment increased seed germination of eight species, suggesting that seed germination is sensitive to atmospheric N deposition in this semi‐arid grassland. Compared to responses of seed germination to N deposition in pot and in situ experiments, our results may provide a new perspective for the study of the reduction of diversity by N deposition. The indirect effect of nitrogen deposition on seed germination was greater or even opposite than the direct effect of nitrogen in pot and in situ studies. Further studies on the direct and indirect effects of N deposition on seed germination are necessary to provide more comprehensive insight into ecological mechanisms structuring these communities.

## CONFLICT OF INTEREST

None declared.

## AUTHOR CONTRIBUTION

**Tong Zhang:** Funding acquisition (equal). **Mengzhou Liu:** Formal analysis (equal); Methodology (equal). **Xudong Huanag:** Formal analysis (equal); Funding acquisition (lead). **Wei Hu:** Funding acquisition (supporting). **Ning Qiao:** Investigation (supporting). **Hongquan Song:** Investigation (equal). **Bing Zhang:** Conceptualization (equal); Data curation (equal); Formal analysis (equal); Investigation (equal). **Rui Zhang:** Funding acquisition (supporting). **Zhongling Yang:** Data curation (equal); Funding acquisition (supporting). **Yinzhan Liu:** Conceptualization (equal); Investigation (equal). **Yuan Miao:** Data curation (equal). **Shijie Han:** Formal analysis (equal). **Dong Wang:** Conceptualization (lead); Data curation (supporting); Formal analysis (supporting); Funding acquisition (supporting); Investigation (supporting); Methodology (supporting); Project administration (supporting).

## Data Availability

A copy of the data will be archived using the DRYAD international repository (https://doi.org/10.5061/dryad.w3r2280nd).
